# Mapping of Antibody Epitopes on the Crimean-Congo Hemorrhagic Fever Virus Nucleoprotein

**DOI:** 10.3390/v14030544

**Published:** 2022-03-06

**Authors:** Boniface Pongombo Lombe, Takeshi Saito, Hiroko Miyamoto, Akina Mori-Kajihara, Masahiro Kajihara, Masayuki Saijo, Justin Masumu, Takanari Hattori, Manabu Igarashi, Ayato Takada

**Affiliations:** 1 Division of Global Epidemiology, International Institute for Zoonosis Control, Hokkaido University, Sapporo 001-0020, Japan; bonifacelombe@czc.hokudai.ac.jp (B.P.L.); t.saito@czc.hokudai.ac.jp (T.S.); hirom@czc.hokudai.ac.jp (H.M.); akinam@czc.hokudai.ac.jp (A.M.-K.); kajihara@czc.hokudai.ac.jp (M.K.); hattoritakanari@czc.hokudai.ac.jp (T.H.); igarashi@czc.hokudai.ac.jp (M.I.); 2 Central Veterinary Laboratory of Kinshasa, Kinshasa B.P. 8842, Democratic Republic of the Congo; justinjmasumu@hotmail.com; 3 Faculty of Veterinary Medicine, National Pedagogic University, Kinshasa B.P. 8815, Democratic Republic of the Congo; 4 Department of Virology 1, National Institute of Infectious Diseases, Musashimurayama 208-0011, Japan; saijo_family@icloud.com; 5 National Institute of Biomedical Research, Kinshasa B.P. 1197, Democratic Republic of the Congo; 6 International Collaboration Unit, International Institute for Zoonosis Control, Hokkaido University, Sapporo 001-0020, Japan; 7 Department of Disease Control, School of Veterinary Medicine, The University of Zambia, Lusaka 10101, Zambia

**Keywords:** Crimean-Congo hemorrhagic fever virus, CCHFV, Nairobi sheep disease virus, nucleoprotein, antibody, epitope, monoclonal antibody

## Abstract

Crimean-Congo hemorrhagic fever virus (CCHFV), a nairovirus, is a tick-borne zoonotic virus that causes hemorrhagic fever in humans. The CCHFV nucleoprotein (NP) is the antigen most used for serological screening of CCHFV infection in animals and humans. To gain insights into antibody epitopes on the NP molecule, we produced recombinant chimeric NPs between CCHFV and Nairobi sheep disease virus (NSDV), which is another nairovirus, and tested rabbit and mouse antisera/immune ascites, anti-NP monoclonal antibodies, and CCHFV-infected animal/human sera for their reactivities to the NP antigens. We found that the amino acids at positions 161–320 might include dominant epitopes recognized by anti-CCHFV IgG antibodies, whereas cross-reactivity between anti-CCHFV and anti-NSDV antibodies was limited. Their binding capacities were further tested using a series of synthetic peptides whose sequences were derived from CCHFV NP. IgG antibodies in CCHFV-infected monkeys and patients were reactive to some of the synthetic peptide antigens (e.g., amino acid residues at positions 131–150 and 211–230). Only a few peptides were recognized by IgG antibodies in the anti-NSDV serum. These results provide useful information to improve NP-based antibody detection assays as well as antigen detection tests relying on anti-NP monoclonal antibodies.

## 1. Introduction

Crimean-Congo hemorrhagic fever (CCHF) is an important tick-borne zoonotic disease with wide geographic distribution. Due to its high mortality rate and potential to cause an epidemic, it constitutes a public health threat [1,2]. Currently, there are no approved vaccines or antiviral drugs for CCHF [3]. In anticipation of a potential epidemic event, the WHO Blueprint ranks CCHF as one of the diseases necessitating research and development attention [4,5]. CCHF is caused by the CCHF virus (CCHFV), a member of the genus *Orthonairovirus* in the family *Nairovidae*. Despite the lack of systemic surveillance and the difficulty in predicting its occurrence, CCHF is known to be endemic in Africa, the Balkans, the Middle East, West Asia, and Southern and Eastern Europe [2,6]. Human-to-human transmission of CCHFV is usually uncommon, but community or nosocomial outbreaks occur when proper infection control practices do not take place [7]. Diagnostic methods for CCHFV include virus isolation, which is restricted to the highest containment laboratories (e.g., biosafety level 4), molecular techniques for viral RNA detection, and serological assays for immunoglobulin detection. However, these tests are carried out only in specialized laboratories in a limited number of countries [7,8].

Among CCHFV proteins, the nucleoprotein (NP), which is antigenically conserved among the CCHFV clades, is the most abundant primary antigen detectable during CCHFV infection and is also known to be highly immunogenic [9]. Thus, CCHFV NP serves as the principal antigen for the development of serological assays [10,11,12,13,14,15,16]. Similar to other hemorrhagic fevers, CCHF tends to be reported in remote areas lacking diagnostic capacity, which causes a delay in its clinical recognition, while retrospective serosurveys provide information on past exposure to the virus but not with active infection [7]. Hence, there is a need to develop effective rapid diagnostic tools such as a lateral flow antigen detection kit that can be used for monitoring CCHFV in the field setting to limit its spread and epidemic potential [9,17].

Previously, we and others showed the usefulness of recombinant CCHFV NP antigens in an enzyme-linked immunosorbent assay (ELISA) and an immunofluorescent assay, and its ability for detecting CCHFV NP-specific IgG directed against strains originating from different geographic regions [10,16,18,19,20]. However, it is known that some anti-NP antibodies are cross-reactive to multiple orthonairoviruses in ELISA and Western blotting [16,18,19]. Considering the overlapping distribution of CCHFV-related orthonairoviruses such as Nairobi sheep disease virus (NSDV), also known as Ganjam virus in Asia, and Dugbe virus (DUGV), both of which are known as zoonotic pathogens, the cross-reactivity of anti-NP antibodies is an important issue for serological diagnosis and surveillance of CCHF [18,19].

In general, mapping of antigenic sites on viral proteins is important for therapeutic, diagnostic, and vaccine development [21,22,23,24]. The identification and characterization of CCHFV NP-specific epitopes will be useful for generating a modified CCHFV NP antigen that reduces the cross-reactivity or producing CCHFV NP-specific monoclonal antibodies that can be used for antigen detection assays. Several previous studies mapped a conserved and immunodominant region on the CCHFV NP molecule, which might be an attractive target for pan-CCHFV diagnosis [9,10,25,26]. To date, however, it is unknown whether some epitopes in this region are common among other nairoviruses, including NSDV and DUGV, and potentially induce cross-reactive antibodies [16,19]. We, therefore, sought to identify the epitopes on the CCHFV NP molecule, using anti-CCHFV NP antisera, CCHFV-infected monkey serum, CCHFV patient sera, immune ascites fluids to the NSDV and DUGV, and anti-CCHFV NP monoclonal antibodies (mAbs).

## 2. Materials and Methods

### 2.1. Construction of Plasmids Expressing CCHFV and NSDV NPs

**Figure 1:**
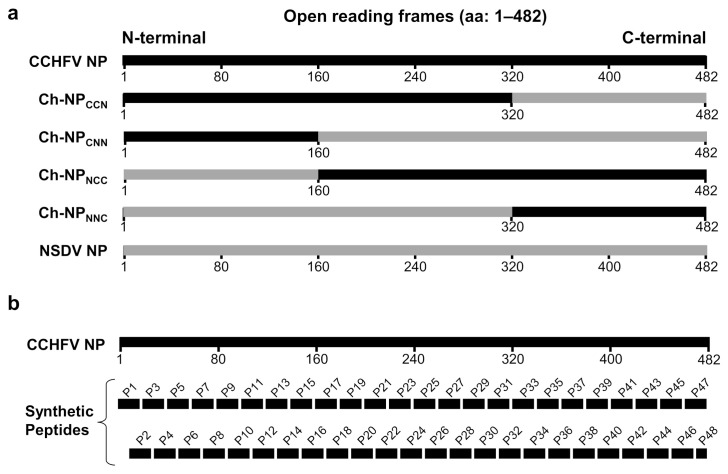
Schematic diagrams of CCHFV and NSDV NPs, their chimeric proteins, and CCHFV NP-based synthetic peptides (**a**) An illustration representing the entire CCHFV (black) and NSDV (gray) NPs and constructed chimeric NPs (Ch-NPs CCN, CNN, NCC, and NNC) is shown. Amino acid positions are indicated below each bar. (**b**) Design and numbers (P1–P48) of 20 amino acid peptides overlapping by 10 amino acids with adjacent peptides, except P48 (12 amino acids) spanning the entire CCHFV NP.

A mammalian expression plasmid, pCAGGS, containing full-length NP genes of CCHFV strain IbAr10200 or NSDV isolate Jilin (GenBank Accession numbers KY484036.1 and NC_034386.1, respectively) was constructed as described previously [16]. Briefly, the CCHFV NP and NSDV NP genes were polymerase chain reaction (PCR)-amplified using viral RNA and synthesized [16], respectively, and cloned into the multiple cloning site of the pCAGGS vector using EcoRI and XhoI restriction sites. Plasmids encoding a series of chimeric NP genes between CCHFV and NSDV (Ch-NPs CCN, CNN, NCC, and NNC) were also constructed in an interwoven fashion (Figure 1a). Briefly, designed fragments were PCR-amplified with a KOD One^TM^ PCR master mix (Toyobo, Osaka, Japan) according to the manufacturer’s protocol using CCHFV and NSDV NP-specific primers designed for further infusion multi-insert cloning (Takara Bio, Shiga, Japan). Amplified gene fragments were gel-purified (Wizard SV Gel and PCR Clean-Up System, Promega) and cloned into the pCAGGS plasmid using an In-Fusion^®^ HD Multi-Insert Cloning Kit (Takara Bio, Shiga, Japan). The sequences of the chimeric NP genes were confirmed using a 3500xL Genetic Analyzer (Hitachi/Applied Biosystems, Tokyo, Japan).

### 2.2. Expression and Purification of NPs

The recombinant NP antigens were generated and purified as previously described [16]. Briefly, human embryonic kidney 293T (HEK293T) cells were transfected with the pCAGGS plasmids encoding NPs, using TransIT-LT1 Transfection Reagent (Mirus Bio LLC, Madison, WI, USA) according to the manufacturer’s protocol. After 48-h incubation, the cells were harvested by pipetting, washed with phosphate-buffered saline (PBS), and treated with lysis buffer (10 mM Tris-HC_l_, pH 7.8, 0.15 M NaCl, 1.0 mM EDTA, and 0.25% NP-40) in the presence of halt protease inhibitor single use cocktail (Thermo Scientific, Waltham, MA, USA), and recombinant NPs were purified from the cell lysate through ultracentrifugation with 20%–50% (*w*/*v*) discontinuous CsC_l_ gradients. The NP fractions were pooled, and purified NP was collected by ultracentrifugation. The concentration of NP was determined using the bicinchoninic acid assay (Pierce Micro BCA Assay Kit-Perce) (Thermo Scientific, Rockford, IL, USA) according to the company instructions and used for enzyme-linked immunosorbent assay (ELISA) and immunization of mice.

### 2.3. Serum and Mouse Immune Ascites Fluids (MIAFs) Samples

Rabbit and mouse polyclonal antisera raised against recombinant CCHFV NP (strains 8402 and IbAr10200, respectively), CCHFV (strain Hoti)-infected monkey, and 5 laboratory-confirmed CCHFV-infected patient serum samples that were used in this study, have been described previously [10,16]. MIAFs to NSDV and DUGV were obtained from the World Reference Center for Emerging Viruses and Arboviruses (WRCEVA), University of Texas Medical Branch, Galveston, TX, USA.

### 2.4. Production and Characterization of Mouse mAbs to CCHFV NP

Anti-NP mAbs were prepared as described previously [23,27]. Briefly, 5-week-old inbreed female BALB/c mice were immunized twice by intraperitoneal injection with 40–50 μg of purified CCHFV NP at 28-day intervals, followed by an intravenous booster immunization with 100 μg of CCHFV NP 6 months after the second immunization. Three days after the booster, spleen cells were collected and splenocytes of the immunized mice were fused with P3-U1 mouse myeloma cells using polyethylene glycol 1500. Hybridomas were selected in medium supplemented with hypoxanthine aminopterin thymidine and hypoxanthine thymidine. The supernatants of hybridomas were screened for the presence of anti-CCHFV NP mAbs in ELISA using cell lysates of transfected HEK293T cells expressing CCHFV, NSDV, and Ch-NPs or purified protein antigens. The cells producing CCHFV NP-specific mAbs were then cloned by a limiting dilution method [23,28]. Anti-NP mAbs were purified from culture supernatants using an Affi-Gel Protein A MAPS kit (BIO-RAD, Hercules, CA, USA) and their isotypes were determined using a mouse monoclonal antibody isotyping test kit, Mouse Isotyping kit (MMT1) (BIO-RAD, Hercules, CA, USA), according to the manufacturer’s instructions. All animal experiments were conducted in strict accordance with the Guidelines for Proper Conduct of Animal Experiments of the Science Council of Japan. The protocol was approved by the Animal Care and Use Committee of Hokkaido University on 30 March 2018 (#18-0029).

### 2.5. ELISA for mAb Screening

ELISA was performed as described previously [16,23]. Briefly, cell lysates (1:1000 dilution) of HEK293T cells transfected with NP-expressing pCAGGS or purified NPs (10 µg/mL) diluted in PBS were used as antigens. Flat-bottom 96-well Nunc Maxisorp ELISA plates (Thermo Fisher Scientific, Waltham, MA, USA) were coated with the antigens followed by blocking with 3% skim milk in PBS. After washing once with PBS containing 0.05% Tween 20 (PBST), 50 μL of culture supernatants of hybridomas were added. After washing with PBST 3 times, the plates were incubated with a secondary antibody; horseradish peroxidase (HRPO)-conjugated goat anti-mouse IgG (H + L) (Jackson ImmunoResearch, West Grove, PA, USA) appropriately diluted with PBST containing 1% skim milk. After washing with PBST 4 times, the bound antibodies were visualized with 50 μL of 3, 3′, 5, 5′-tetramethylbenzidine (TMB) substrate (Sigma, St. Louis, MO, USA). The reaction was stopped by adding 1 N phosphoric acid and the optical density (OD) at 450 nm was measured using a SpectraMAX 190 device (Molecular Devices, San Jose, CA, USA).

### 2.6. Western Blotting

The lysates prepared from HEK293T cells transfected with pCAGGS expressing NPs were subjected to SDS-PAGE under reducing conditions (β-mercaptoethanol). Separated proteins were transferred onto a polyvinylidene membrane, Immobilon-P (Merck Millipore Corporation, Burlington, MA, USA), followed by blocking with 3% skim milk in PBS. Rabbit antiserum to CCHFV NP, mouse antiserum to CCHFV NP, MIAFs to NSDV and DUGV, CCHFV-infected monkey serum, CCHFV patient sera, and purified mAbs were diluted at 1:2500, 1:1000, 1:2500, 1:10,000, 1:1000, and 2 μg/mL, respectively with PBST containing 1% skim milk, and used as primary antibodies. HRPO-conjugated goat anti-rabbit IgG (H + L) (KPL, Gaithersburg, MD, USA), goat anti-mouse IgG (H + L) (Jackson ImmunoResearch, West Grove, PA, USA), goat anti-monkey IgG(γ) (Rockland, Limerick, PA, USA), and goat anti-human IgG (H + L) (Jackson ImmunoResearch, West Grove, PA, USA) were appropriately diluted with PBST containing 1% skim milk and used as secondary antibodies, and reactions were visualized with Immobilon Western chemiluminescent HRPO substrate (Merck Millipore Corporation, Burlington, MA, USA).

### 2.7. ELISA with Synthetic Peptides as Antigens

A library of 48 synthetic peptides spanning the entire CCHFV NP sequence of strain IbAr10200 was designed as described previously and produced by PEPscreen custom peptide libraries (Sigma, St. Louis, MO, USA) [28]. Each peptide had 20 amino acids including 10 overlapping residues with adjacent peptides except peptide 48 (12 amino acids) (Figure 1b). The synthetic peptides were reconstituted in dimethyl sulfoxide (DMSO) (FUJIFILM Wako Pure Chemical Corporation, Osaka, Japan) at a final concentration of 2 mg/mL. The reactivities of antibodies to synthetic peptides were assessed in ELISA. Flat-bottom 96-well Maxisorp ELISA plates were coated overnight at 4 °C with 50 μL of carbonate buffer (pH 8.0) containing 100 or 200 µg/mL of each peptide. The peptide solution was then replaced with 180 µL of 3% skim milk in PBS for blocking for 1 h. After washing once with PBST, the plate was incubated with appropriately diluted antibodies (i.e., mAbs, CCHFV-infected patient, and monkey serum samples, or MIAFs to NSDV and DUGV) for 1 h at RT. The plate was washed 3 times with PBST and then incubated with corresponding secondary antibodies as described above or HRPO-conjugated protein A/G (Thermo Fisher Scientific, Rockford, IL, USA). The bound antibodies were visualized with the TMB substrate and OD values were measured, as described above.

### 2.8. Structural Analysis

The structural data of the CCHFV NP molecule were obtained from Protein Data Bank (PDB) (PDB ID 3U3I). The missing amino acid residues at positions 182–194, 243, and 367–371 were added using Molecular Operating Environment (MOE) software (version 2018; Chemical Computing Group, Montreal, QC, Canada). The three-dimensional structure of the molecule was generated using PyMOL software version 4.6.0 (Schrödinger, New York, NY, USA).

### 2.9. Amino Acid Sequence Analysis of Cross-Reactive Peptides

To compare the amino acid sequences of the identified cross-reactive peptide regions, the NP amino acid residues of CCHFV strain IbAr10200 (ARB51456.1), NSDV strain 6233 (AEK67471.1), and DUGV strain Ib Ar 1792 (AMT75394.1) were retrieved from GenBank at the National Center for Biotechnology Information (NCBI). Multiple sequence alignment was performed using the MUSCLE program through MEGA software version 10.2.2.

### 2.10. Statistical Analysis

To determine the statistical significance in ELISA with synthetic peptides, the OD values obtained from PEPscreen reactions were assumed to follow the normal distribution. For CCHFV-infected patients and monkey, the threshold defining positive reaction was obtained based on the average + 3 × standard deviation of all the OD values (P1-P48) of negative control (normal) serum samples. Since MIAF-negative control samples were not available, the Smirnov–Grubbs rejection test, which is widely used to detect outliers that do not belong to the population consisting of all other values in the data set, was employed for the MIAFs to the NSDV and DUGV. Briefly, if the highest OD value was considered to be an outlier, the *T* value for the second-highest OD value was similarly tested without the highest one. These steps were repeated until the *T* value fell to below the level of statistical significance (*p* < 0.01).

### 2.11. Ethics Statement

All animal experiments were conducted in strict accordance with the Guidelines for Proper Conduct of Animal Experiments of the Science Council of Japan under approval (#18-0026) by the Institutional Animal Care and Use Committee (Hokkaido University). The use of human serum samples was approved by the medical research ethics committee of the National Institute of Infectious Diseases for the use of human subjects, Tokyo, Japan (No. 10).

## 3. Results

### 3.1. Reactivity of Antisera/MIAF and CCHFV-Infected Monkey/Human Sera to CCHFV and NSDV Chimeric NPs in Western Blotting

CCHFV and NSDV NP fragments were joined in an interwoven fashion in the pCAGGS plasmid. The chimeric proteins, Ch-NPs (CCN, CNN, NCC, and NNC), gradually had 160–162 amino acid sequences of CCHFV NP from the N- to C-terminal while deleting those of NSDV NP and vice versa (Figure 1a). HEK293T cells were transfected with each plasmid encoding NPs, and the cell lysates were used for Western blotting. All the chimeric proteins were expressed in the cells and antisera/MIAF and CCHFV-infected monkey sera were tested for their reactivities to wildtype CCHFV NP, NSDV NP, and Ch-NPs in Western blotting (Table 1). Anti-CCHFV NP rabbit antiserum, CCHFV-infected monkey serum, and anti-CCHFV NP mouse serum all reacted to CCHFV NP, Ch-NPs CCN, and NCC but not to NSDV NP, Ch-NPs CNN and NNC. In contrast, anti-NSDV serum reacted to NSDV NP, Ch-NPs CNN, and NNC, whereas CCHFV NP and the other Ch-NPs were almost undetectable in this serum. As well as anti-NSDV serum, anti-DUGV serum predominantly reacted to NSDV NP, Ch-NPs CNN, and NNC. Interestingly, however, this serum showed a little cross-reactivity to CCHFV NP. CCHFV-infected patient serum samples showed reaction patterns similar to those of anti-CCHFV NP rabbit antiserum and anti-CCHFV NP mouse serum (i.e., they were generally reactive to CCHFV NP, Ch-NPs CCN and NCC but not to NSDV NP and the other Ch-NPs) (Table 1). Taken together, these results suggested that the overall antigenicity was not similar between CCHFV and NSDV NPs, and that amino acids at positions 161–320 of both NPs included dominant epitopes recognized by anti-NP IgG antibodies.

### 3.2. Reactivity of Anti-NP mAbs in Western Blotting

To further obtain information on the antigenic regions on NP, we then generated 12 mouse mAbs reactive to CCHFV NP, as described in materials and methods. The majority of the generated mAbs were IgG2a and IgG2b with a light chain kappa. These mAbs reacted to purified CCHFV NP but not to NSDV NP in ELISA. Based on the reactivity pattern to a series of the NP antigens, the 12 mAbs were separated into 3 groups (Table 2). Three mAbs in group I did not react to CCHFV NP. None of the generated mAbs recognized NSDV NP in Western blotting, whereas CCHFV NP was recognized by 9 of the 12 mAbs (groups II and III). Of the mAbs reacting to CCHFV NP in Western blotting, four mAbs (32-1, 74-2, 86-3, and 114-2) recognized none of the Ch-NPs. On the other hand, five mAbs (17-3, 79-10, 80-6, 91-5, and 97-6) recognized Ch-NPs CCN and NCC. Reactivities of the five mAbs (17-3, 79-10-3, 80-6, 91-5, and 97-6) to the Ch-NPs suggested that these mAbs recognized epitopes on amino acid positions 161–320 of CCHFV NP.

### 3.3. Identification of Epitope Peptides on the CCHFV NP Molecule in ELISA

**Figure 2:**
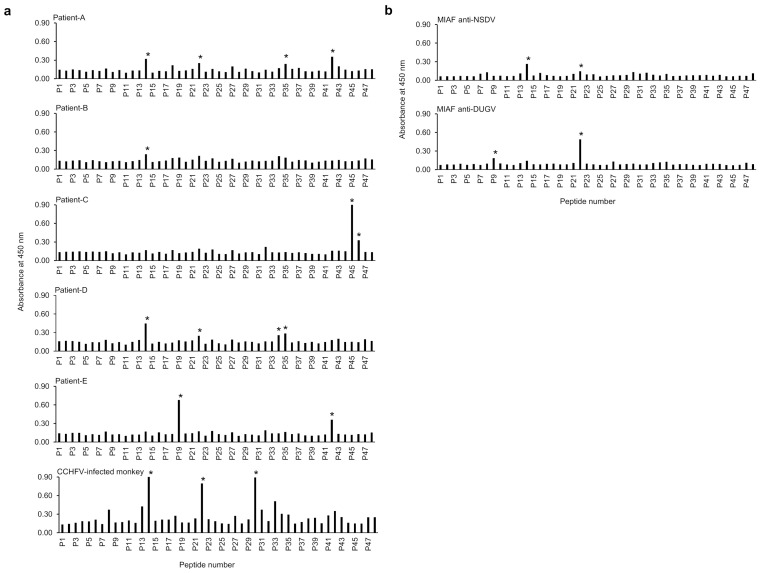
Reactivities of polyclonal antibodies to the PEPscreen library of CCHFV NP. The peptides are numbered according to their positions (see also Figure 1 and Table 2). (**a**) The serum samples of CCHF patients and a CCHFV-infected monkey or MIAFs to NSDV and DUGV (**b**) were diluted at 1:500 and used as primary antibodies. Experiments were duplicated, and the averages are shown. Significantly higher OD values are indicated by asterisks.

**Figure 3:**
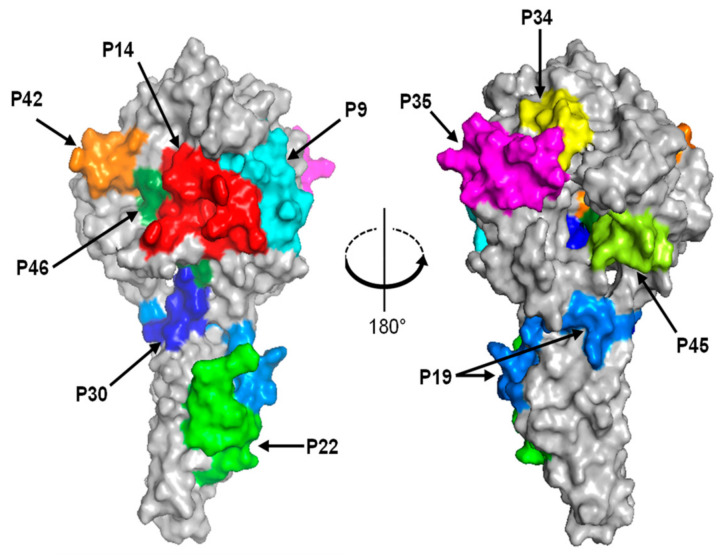
Mapping of the identified epitope regions on the CCHFV NP molecule. The monomeric structure of the CCHFV NP molecule is shown as a surface model. Amino acid locations in the 3D structure of the 10 peptides shown in Table 3 are depicted in distinct colors.

Next, we tried to determine CCHFV NP-specific epitopes using a synthetic peptide library based on the NP sequence of CCHFV strain IbAr10200 (Figure 1b). Forty-eight peptides were used as antigens in ELISA. To focus on epitopes for naturally induced antibodies, CCHF patient and CCHFV-infected monkey sera were tested for IgG antibodies reactive to each peptide. We found that the reactive peptides were scattered from the N- to C-terminals over the length of the CCHFV NP sequence and the reaction patterns varied among individuals (Figure 2a and Table 3). Synthetic peptides P14 and P22, corresponding to the amino acid residues at positions 131–150 and 211–230, respectively, were recognized by IgG antibodies in multiple patient and infected monkey sera. Two peptides (P35 and P42) also gave a positive reaction for two of the five patient sera. Some other peptides (P19, P30, P34, P45, and P46) were recognized by IgG antibodies in either patient or monkey sera. Of these, the amino acid positions of P19, P22, and P30 peptides were consistent with the fragment recognized by NP-specific IgG antibodies in Western blotting shown in Table 1 (i.e., amino acid positions 161–320). In contrast, all the CCHFV NP mAbs generated in this study failed to recognize these synthetic peptides. To further assess the existence of conserved epitopes among CCHFV, NSDV, and DUGV NPs, MIAF to NSDV and DUGV were also tested in ELISA using the PEPscreen library (Figure 2b and Table 3). We found that P9 and P14 peptides were recognized by MIAFs to DUGV and NSDV, respectively, and P22 was recognized by both MIAFs. We further confirmed that amino acid regions corresponding to these 10 peptide sequences were exposed on the molecular surface of CCHFV NP (Figure 3).

### 3.4. Amino Acid Sequence Comparison for the Common Epitope Peptides

**Figure 4:**
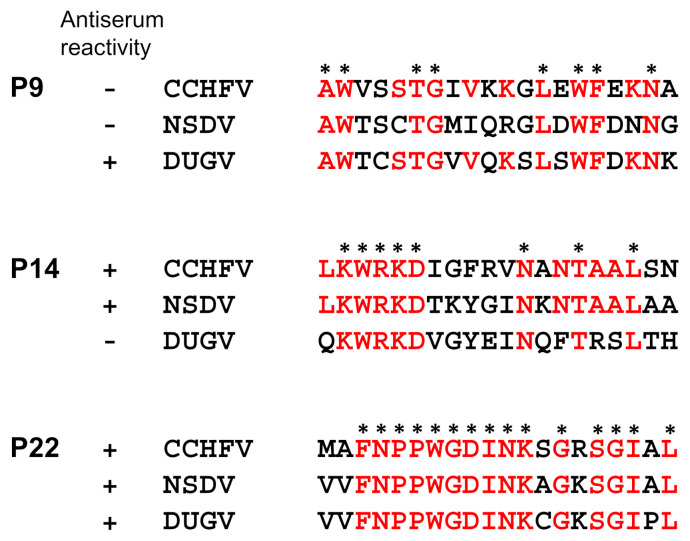
Amino acid sequence comparison among CCFHV, NSDV, and DUGV NPs. Amino acid sequences of CCHFV, NSDV, and DUGV NPs corresponding to the P9, P14, and P22 peptides are shown. Amino acid residues conserved among all three viruses are indicated with asterisks. Amino acid residues shared between CCHFV and DUGV NPs, between CCHFV and NSDV NPs, and among all these NPs are shown in red for P9, P14, and P22, respectively.

The amino acid sequences of the peptides that were commonly recognized by IgG antibodies to CCHFV, NSDV, and/or DUGV (P14, and P22), and the peptide recognized uniquely by IgG to DUGV (P9), were compared among CCHFV, NSDV, and DUGV NPs (Figure 4). As expected, these peptides had relatively conserved amino acid sequences among the viruses. The amino acid sequence identity of P9 between CCHFV and DUGV was higher (60%) than those between CCHFV and NSDV (50%). The sequence identity of P14 between CCHFV and NSDV was 60%, whereas that between CCHFV and DUGV was 45%. These differences were correlated with the cross-reactivity of the MIAFs to the respective peptides. The P22 peptide, to which anti-CCHFV, -NSDV, and -DUGV IgG antibodies all bound in ELISA, had 75% (15/20) conserved amino acid residues, which was also consistent with cross-reactivity of the antibodies in ELISA. These data suggested that the amino acid similarities of these peptides among CCHFV, NSDV, and DUGV were correlated with the serological cross-reactivity.

## 4. Discussion

Antibodies to CCHFV NP are known to potentially cross-react to other orthonairoviruses [16,18,19]. Although CCHFV NP epitopes have been mapped in previous studies [9,25,26,29], detailed information on the epitopes on orthonairoviruses is still needed. To discern and identify antigenic regions on CCHFV and NSDV NPs, CCHFV-NSDV chimeric NPs were produced and used for Western blotting analyses in this study. We found that IgG antibodies in antisera/MIAF produced against CCHFV were mostly bound to Ch-NPs CCN, and NCC, whereas those against NSDV and DUGV were bound to Ch-NPs CNN, and NNC. These results suggest that there may be a limited number of conserved epitopes among these viruses. Our Western blotting analysis further suggests that CCHFV- and NSDV-specific epitopes are located in the amino acid regions at positions 161–320, which is the overlapping region of the respective chimeric proteins. Consistent with these data of polyclonal antibodies to CCHFV, none of the 12 mAbs generated in this study reacted to NSDV NP and 5 mAbs (17-3, 79-10-3, 80-6, 91-5, and 97-6) reacted to Ch-NPs CCN, and NCC, suggesting that these 5 mAbs recognized the amino acid region at positions 161-320. Four mAbs (32-1, 74-2, 86-3, and 114-2) reacted to wildtype CCHFV NP and did not react to any chimeric NPs tested, suggesting that these mAbs recognized epitopes different from those of the other five mAbs that reacted to Ch-NPs CCN, and NCC. Overall, these results demonstrate the presence of at least three distinct antigenic regions on the CCHFV NP molecule.

Previous papers by others have demonstrated that the amino acid region at positions 183–305 of CCHFV NP contains major antibody epitopes [9,10,25,26,30]. However, the N- and C-termini of NP have also been shown to include some epitopes [26,29]. Consistent with the results of these studies, the polyclonal and monoclonal antibodies used in our study reacted to the amino acid regions including positions 161–320 of CCHFV NP. PEPscreen library-based ELISA to further identify the epitope sequences revealed at least 10 epitope peptides scattered along the length of the CCHFV NP molecule. Distinct antibody repertoires among CCHFV-infected individuals were also suggested, consistent with previous studies [26,30]. The epitope peptides for CCHFV-specific IgG were located in a wide region of CCHFV NP spanning amino acid positions from 131 to 470, which overlapped with the previously reported region consisting of amino acid residues at positions 123 to 396 [26]. However, our approach (i.e., Western blotting and short peptide antigens) has a limitation in that conformational epitopes cannot be identified.

None of the generated anti-NP mAbs bound to the synthetic peptides tested in this study. This probably indicates that they recognize conformational epitopes. Although some of the mAbs reacted to CCHFV NP in Western blotting, it is conceivable that the synthetic peptides used (20 amino acid-length) might not be able to form proper structures. It has also been shown that a predicted biosynthetic CCHFV NP linear antibody epitope expressed in *E. coli* (DH5α) is not recognized by mAbs or polyclonal anti-CCHFV [25,31]. Since these antibodies showed no cross-reactivity to NSDV NP, it is likely that they are CCHFV NP-specific. Structural analyses using an antibody-NP complex will be required for further detailed epitope mapping of CCHFV NP.

Although the genetic diversity among nairovirus NPs is significant [32], the viruses within NSDV and CCHFV groups are closely related [33,34]. Previously, a linear epitope was predicted within the P22 sequence region of CCHFV NP (strain SPU 415/85) and the antigenic similarity between CCHFV and DUGV has been reported [9,26,33]. The present study also pointed out the sequence similarity in some of the peptide sequences among CCHFV, NSDV, and DUGV, suggesting the potential cross-reactivity of antibodies to orthonairovirus NPs of the same serogroup. Importantly, however, our data suggest that although there are some common epitopes between NSDV/DUGV and CCHFV, such cross-reactive epitopes are not dominant as indicated by little cross-reactivity of the respective antibodies in Western blotting.

In this study, we focused on antibody epitopes on CCHFV NP and the presence of shared epitopes with NSDV/DUGV NPs. However, it is also important to analyze the cross-reactivities of antibodies to other nairoviruses closely related to CCHFV, such as Hazara, Tofla, Meram, and Kupe viruses, while their pathogenic potential to humans is unclear [32,35,36,37,38,39,40]. Although several serological diagnostic assays for CCHF have been previously established, the cross-reactivity issue is still a potential weakness of the assays since the whole NP molecule is used as an antigen. Fine epitope mapping of CCHFV NP is expected to improve the serological methods that can be used to establish a rapid point-of-care detection assay. In addition, the mAbs produced in this study are useful for the development of immunochromatography-based rapid diagnostic tests.

## Figures and Tables

**Table 1:** Reactivities of anti-CCHFV serum and MIAF to NPs in Western blotting ^a^.

Serum and MIAF	CCHFV NP	Ch-NP				NSDV NP
		CCN	CNN	NCC	NNC	
Rabbit anti-CCHFV NP	+	+	−	+	−	−
Mouse anti-CCHFV NP	+	+	−	+	−	−
Mouse anti-NSDV	−	−	+	−	+	+
Mouse anti-DUGV	± ^c^	−	+	−	+	+
CCHFV-infected monkey	+	ND ^b^	ND	ND	ND	−
CCHFV-infected patient #A	+	+	−	+	−	−
CCHFV-infected patient #B	+	+	−	+	−	−
CCHFV-infected patient #C	+	−	± ^c^	−	−	−
CCHFV-infected patient #D	+	+	−	+	−	−
CCHFV-infected patient #E	+	+	−	+	−	−

^a^ Western blotting was performed using lysates of HEK293T cells transfected with NP-expressing plasmids under reducing conditions. ^b^ Not determined due to the high background and nonspecific reactions. ^c^ Band weakly seen.

**Table 2:** Classification of mouse anti-CCHFV NP mAbs based on their reactivities in Western blotting.

Group	mAb	ELISA (CCHFV NP) ^b^	CCHFV NP ^a^	Ch-NP ^a^				NSDV NP ^a^	Isotype
				CCN	CNN	NCC	NNC		
I	09-2	+	−	−	−	−	−	−	IgG2b kappa
	27-6	+	−	−	−	−	−	−	IgG2b kappa
	87-5	+	−	−	−	−	−	−	IgG2b kappa
II	32-1	+	+	−	−	−	−	−	IgG2b kappa
	74-2	+	+	−	−	−	−	−	IgG2b kappa
	86-3	+	+	−	−	−	−	−	IgG2b kappa
	114-2	+	+	−	−	−	−	−	IgG2b kappa
III	17-3	+	+	+	−	+	−	−	IgG2b kappa
	79-10	+	+	+	−	+	−	−	IgG2a kappa
	80-6	+	+	+	−	+	−	−	IgG2a kappa
	91-5	+	+	+	−	+	−	−	IgG2a kappa
	97-6	+	+	+	−	+	−	−	IgG2a kappa

^a^ Western blotting was performed using lysates of HEK293T cells transfected with NP-expressing plasmids under reducing conditions. ^b^ Purified NP was used as an antigen.

**Table 3:** Synthetic peptides to which anti-NP antibodies in the sera and MIAFs bound.

Peptide Name (Amino Acid Position)	CCHFV-Infected Patient					CCHFV-Infected Monkey	MIAF	
	A	B	C	D	E		NSDV	DUGV
P9 (81–100)	−	−	−	−	−	−	−	+
P14 (131–150)	+	+	−	+	−	+	+	−
P19 (181–200)	−	−	−	−	+	−	−	−
P22 (211–230)	+	−	−	+	−	+	+	+
P30 (291–310)	−	−	−	−	−	+	−	−
P34 (331–350)	−	−	−	+	−	−	−	−
P35 (341–360)	+	−	−	+	−	−	−	−
P42 (411–430)	+	−	−	−	+	−	−	−
P45 (441–460)	−	−	+	−	−	−	−	−
P46 (451–470)	−	−	+	−	−	−	−	−

## Data Availability

Not applicable.
